# A Novel Simulated Annealing Based Strategy for Balanced UAV Task Assignment and Path Planning

**DOI:** 10.3390/s20174769

**Published:** 2020-08-24

**Authors:** Lisu Huo, Jianghan Zhu, Guohua Wu, Zhimeng Li

**Affiliations:** 1 College of Systems Engineering, National University of Defense Technology, Changsha 410073, China; huolisu09@nudt.edu.cn (L.H.); jhzhu72@gmail.com (J.Z.); zmli@nudt.edu.cn (Z.L.); 2 School of Traffic and Transportation Engineering, Central South University, Changsha 410075, China

**Keywords:** heuristic algorithm, path planning, simulated annealing, unmanned aerial vehicle

## Abstract

The unmanned aerial vehicle (UAV) has drawn increasing attention in recent years, especially in executing tasks such as natural disaster rescue and detection, and battlefield cooperative operations. Task assignment and path planning for multiple UAVs in the above scenarios are essential for successful mission execution. But, effectively balancing tasks to better excavate the potential of UAVs remains a challenge, as well as efficiently generating feasible solutions from the current one in constrained explosive solution spaces with the increase in the scale of optimization problems. This paper proposes an efficient approach for task assignment and path planning with the objective of balancing the tasks among UAVs and achieving satisfactory temporal resolutions. To be specific, we add virtual nodes according to the number of UAVs to the original model of the vehicle routing problem (VRP), thus make it easier to form a solution suitable for heuristic algorithms. Besides, the concept of the universal distance matrix is proposed to transform the temporal constraints to spatial constraints and simplify the programming model. Then, a Swap-and-Judge Simulated Annealing (SJSA) algorithm is therefore proposed to improve the efficiency of generating feasible neighboring solutions. Extensive experimental and comparative studies on different scenarios demonstrate the efficiency of the proposed algorithm compared with the exact algorithm and meta-heuristic algorithms. The results also inspire us about the characteristics of a population-based algorithm in solving combinatorial discrete optimization problems.

## 1. Introduction

The unmanned aerial vehicle (UAV), as an aircraft without people on the platform but in the system, has obvious advantages compared with normal aircraft in case of dull dirty and dangerous missions [1]. The use of multiple UAVs in disaster scenarios can provide support for decision-making by government or rescue teams. In this kind of application scenarios, UAVs are usually utilized for taking pictures of disaster areas to update information instantly, and sometimes for delivering necessities for people trapped in an unfavorable environment where general vehicles cannot reach. UAVs have also been widely used for providing information coverage as well as relay service for ground Internet of Things (IoT) networks, in which UAVs can act as mobile edge computing nodes to improve the quality of service (QoS) of IoT nodes or quality of experience (QoE) for users [2,3]. Advantage of using UAVs instead of traditional vehicles is that they are not limited by established infrastructure such as roads, and generally face less complex obstacle avoidance scenarios [4]. This makes UAVs especially suitable for emergency reconnaissance and rescue in natural disasters such as earthquakes and forest fires.

In the above application scenarios, task assignment and path planning are fundamental problems for UAVs to complete tasks efficiently. The general path planning problem for UAVs can be formulated as an optimization problem with multiple constraints, caused by minimal flight length, minimal flight time, and energy consumption [5]. The problem can also be viewed as a three-dimensional (3D) Vehicle Routing Problem (VRP), which is a generalization of the Traveling Salesman Problem (TSP) [6]. In scenarios with multiple target nodes and multiple UAVs, the optimization problems may evolve into large-scale combinatorial optimization problems, which usually require huge computing resources to get optimal solutions.

For the task assignment of multiple UAVs, many methods have been adopted to address this problem. For example, Shima et al. used the genetic algorithm for assigning multiple agents to perform multiple tasks on multiple targets [7]. The genetic algorithm was also used in [8] for multiple UAVs cooperative reconnaissance mission planning problems. Zhao et al. established the task planning model according to task clustering of each UAV and proposed a task allocation method based on improved K-means clustering algorithm of simulated annealing [9]. A hierarchical task assignment method is proposed in [10], which broke the original problem down to sub-problems and solved them with mixed-integer programming and ant colony algorithm. For UAVs with different sensor capabilities, a modified Symbiotic Organisms Search (SOS) algorithm was adopted to optimize UAVs’ task sequence [11]. In article [12], an iterative strategy was proposed to enhance the performance of task assignment and path planning in applications of distributed multiple UAVs. For task allocation problems in a dynamic environment, article [13] proposed a quantum evolutionary inspired algorithm to minimize resource consumption and enhance the reliability of the coalitions of UAVs.

The path planning methods of UAV has attracted even more attention in recent years. Due to the nature of the problem, which usually belongs to the NP-Complete class of problems, population-based meta-heuristic algorithms have been widely used to produce satisfactory solutions. A framework based on Differential Evolution (DE) was utilized to design the off-line path planner for UAVs in [14], and two-dimensional (2D) trajectories were produced by successive way-points while certain coordination constraints were considered. DE was also combined with the quantum-behaved particle swarm optimization (PSO) for UAV route planning in the marine environment [15]. The proposed multiobjective DE in [16] provides a possible solution for UAV path planning in synthetic aperture radar application. Modified or improved DE has also been applied in trajectory following and UAV formation [17]. Other population-based evolutionary algorithms that are frequently used include the Genetic Algorithm (GA), PSO, and their modified versions. Roberge et al. in their study used the GA and PSO to deal with the automatic path planning problem and performed a comparison of these two algorithms which showed that GA produced better trajectories to the PSO [18]. GA has also been used in solving multiobjective mission planning problems involving a group of UAVs and a set of ground control stations [19].

Different from DE and GA, meta-heuristic algorithm Simulated Annealing (SA) is not a population-based approach, but it is still very popular in solving task and path planning problems. For example, SA can be combined with GA to cope with three-dimensional route planning [20,21]. In another case, the K-means algorithm and SA algorithm were combined for problems with multiple UAVs and multiple missions under complicated constraints [22]. 2D path planning in application scenarios with radar threatening was also studied in [23], in which SA algorithm was adopted to obtain nearly optimal path in 2D constrained environment, the author also tried to solve the route planning problems for multiple UAVs by executing parallel SA algorithms in [24], and demonstrated its effectiveness.

The literature discussed above provides some useful methods for task assignment and path planning of UAVs. However, some important issues also urgently need to be addressed. First, many studies still focus on single UAV or 2D application scenarios, while in practical applications, since the load and battery life of single UAV are limited due to the weight constraint, cooperative execution of tasks by multiple UAVs in the 3D environment has become a trend. Second, few studies considered the balanced task distribution and overall temporal resolution in scenarios that need UAVs to perform tasks jointly, such as in the case of natural disaster relief where timely regional information updates are required. Third, for large-scale optimization problems such as task assignment and path planning, due to the large number of decision variables, the solution space could expand exponentially with the increase of variables. Worse yet, a variety of strict constraints lead to a small proportion of feasible solutions in the solution space. Due to the randomness of mutation and crossover operators in general population-based algorithms, they are very likely to just obtain infeasible solutions based on the existing solutions. The conversion process to feasible solutions could greatly increase the time consumption of algorithms. Last but not least, although many heuristic algorithms are used in the task assignment and path planning of UAVs, the effects of different kinds of popular algorithms in solving task assignment and path planning problems have not been analyzed and compared, this is, however, important to provide us with guidance on how to choose different types of algorithms.

In this paper, we propose an efficient heuristic algorithm based on Simulated Annealing, with Swap-and-Judge strategy, namely SJSA, for multiple UAVs task assignment and path planning. By adding virtual nodes to the original 3D VRP model according to the number of UAVs, we encode the feasible solutions for multiple UAVs into a single array. Considering the temporal resolution or maximum time constraints in a mission, we build the task assignment and path planning model, and the balance of task assignment among UAVs is taken into account. Finally, in the context of eight test scenarios and six application scenarios, the proposal is compared with the exact algorithm CPLEX and three representative algorithms, respectively, and the proposed SJSA is demonstrated to be efficient and competitive through experimental and comparative studies.

The remains of this paper is structured as follows: Section 2 formulates the problem, gives the concept of the universal distance matrix and builds the model with the consideration of the temporal resolution and balanced constraints. The algorithms analysis and design is presented in Section 3, in which the population-based approaches are studied and the proposed strategy based on SA are presented. Experiments and comparative studies in different scenarios are detailed in Section 4. Section 5 concludes this paper.

## 2. Problem Formulation and Modeling

In this section, the problem of UAV scheduling with time resolution or maximum flying distance constraints is analyzed. On this basis, the concept of the universal distance matrix is proposed and the model with temporal and balanced constraints is established. Furthermore, the scheme of feasible solution representation and generation is presented.

We consider the UAV application scenario in which a bunch of known target nodes need to be assigned to a group of UAVs, which are going to be programmed to start from depot to visit these targets in limited time and, of course, within the maximum flying distance of each UAV. Another similar scenario is to apply UAVs to periodic sensing mission, in which a group of UAVs need to traverse target nodes and return to the command center with a temporal resolution to guarantee the timely update of the map information.

**Figure 1:**
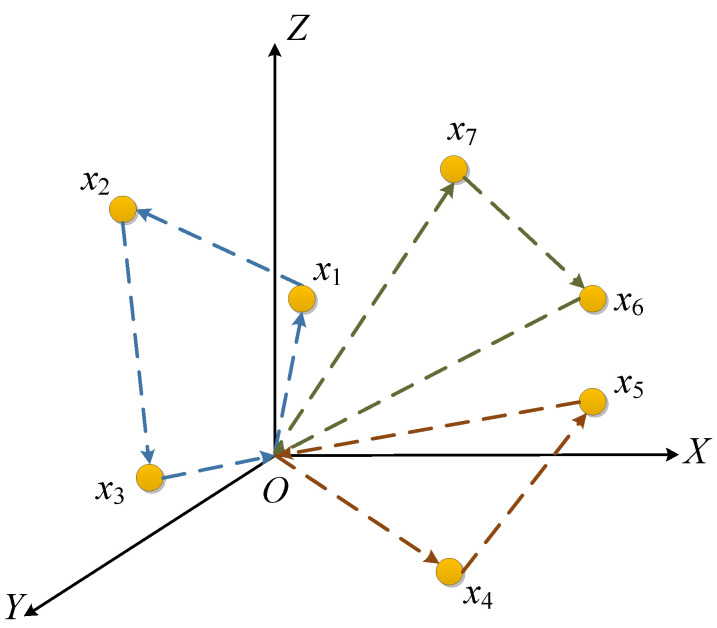
Unmanned aerial vehicle (UAV) task assignment and path planning model in the application scenario.

In above application scenarios, UAVs start from depot *O* to traverse *N* target nodes, like presented in Figure 1, different colors means the generated path for different UAVs, the first UAV is assigned to targets $\left[x_{1},x_{2},x_{3}\right]$ and traverse them in the sequence order, the second UAV is assigned to targets $\left[x_{4},x_{5}\right]$, and the third is assigned to targets $\left[x_{6},x_{7}\right]$ and traverse them in the order $x_{7}\to x_{6}$. After the mission is accomplished, all UAVs return to the original depot directly.

**Figure 2:**
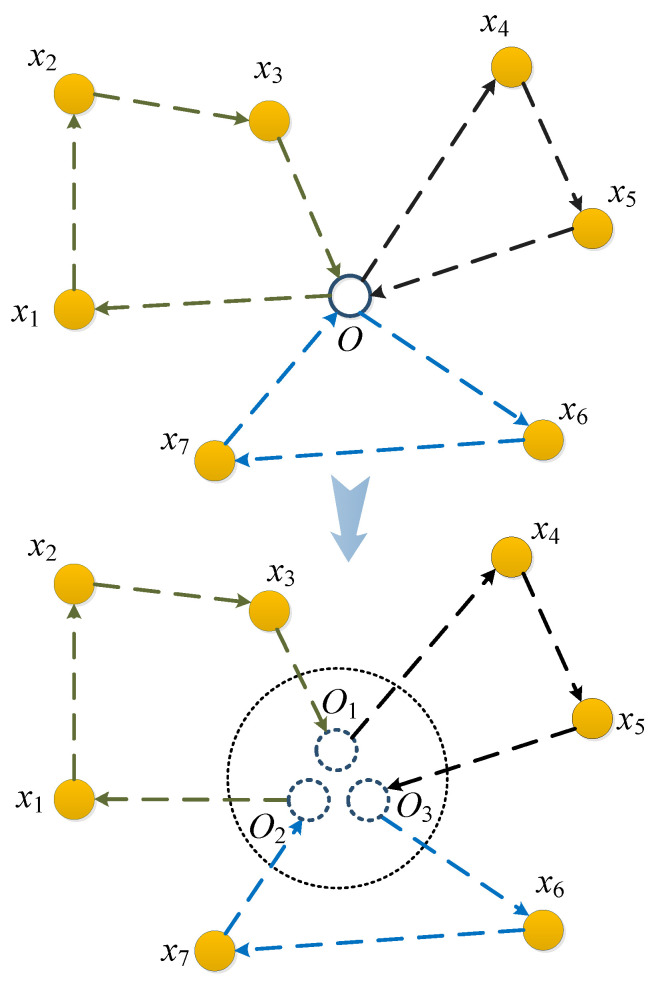
Convert the multipath problem into a single path problem.

Generally, we assume there are $N\_node=\left\{x_{1},x_{2},\ldots ,x_{N}\right\}$ target nodes ready to be visited, and the number of UAV is *M*, considering that every UAV need to return to the depot after its mission finished, we convert the multitrip path generation problem into a single trip for the group of UAVs by duplicating the depot node up to *M* virtual depot nodes, thereby different trips by different UAVs are connected from the first to the last UAV. Like shown in Figure 2, if the objective is only to minimize the total distance of all the path, then the task assignment and path planning problem for multiple UAVs would be similar to the TSP, except that the constraints here are more complicated than that of TSP, which belongs to the NP-Complete class of problems [25].

It is worth mentioning that the target nodes here are not necessarily equal to the task locations. Since UAVs do not have to fly directly above the fixed nodes to perform the task, the actual task locations can be anywhere near the target nodes. For example, in periodic sensing missions where UAVs are required to take pictures to update map information, they can fly above task locations such as architectural or disaster scenes. However, the points which UAVs have to pass by or stay at need to be determined before planning, these points are identified as target nodes. Similarly, UAVs do not necessarily fly straight lines, these straight lines are used to represent the basic flight order and distances among all target nodes, the actual flight distance can be considered and represented by these straight lines before planning.

### 2.1. Universal Distance Matrix

Suppose that $x_{1}$ denotes the depot node or the command center, and $N\_node=\left\{x_{2},x_{3},\ldots ,x_{N+1}\right\}$ are all of the *N* target nodes, the three-dimensional coordinates of all the nodes above are known. All nodes including the depot can be expressed as $V=\left\{x_{1}\right\}\cup \{x_{2},x_{3},\ldots ,x_{N+1}\}=\{x_{i}|i\in \left\{1,2,\ldots ,N+1\right\}\}$, then we can get the distance $s_{ij}$ from any pair $\left(x_{i},x_{j}\right)$ of nodes according to following equation.

(1) $$s_{ij}=\sqrt{{\left(X_{i}-X_{j}\right)}^{2}+{\left(Y_{i}-Y_{j}\right)}^{2}+{\left(Z_{i}-Z_{j}\right)}^{2}},$$

where $\left(X_{i},Y_{i},Z_{i}\right)$ and $\left(X_{j},Y_{j},Z_{j}\right)$ are the 3D coordinates of nodes $x_{i}$ and $x_{j}$. Furthermore, we can get the distance matrix *S* as follows:

(2) $$S=\left[\begin{array}{ccccccc} s_{11} & \ldots & s_{1i} & \ldots & s_{1j} & \ldots & s_{1\left(N+1\right)}\\ \ldots & \ldots & \ldots & \ldots & \ldots & \ldots & \ldots \\ s_{i1} & \ldots & s_{ii} & \ldots & s_{ij} & \ldots & s_{i\left(N+1\right)}\\ \ldots & \ldots & \ldots & \ldots & \ldots & \ldots & \ldots \\ s_{j1} & \ldots & s_{ji} & \ldots & s_{jj} & \ldots & s_{j\left(N+1\right)}\\ \ldots & \ldots & \ldots & \ldots & \ldots & \ldots & \ldots \\ s_{\left(N+1\right)1} & \ldots & s_{\left(N+1\right)i} & \ldots & s_{\left(N+1\right)j} & \ldots & s_{\left(N+1\right)\left(N+1\right)} \end{array}\right].$$

Usually, the diagonal elements of the distance matrix *S* are zeros, and the time of a UAV staying at any node after the visit is accomplished is not considered.

In a practical application scenario, as the target nodes denote different tasks to be executed, the execution time may vary from node to node. Without loss of generality, we propose the concept of universal distance matrix $S_{d}$, which is used to encompass the time delay of nodes. Suppose that the maximum velocity of UAV is $v_{max}$, the time needed to accomplish the task on node $x_{i}$ is $t_{i}$, then the virtual extra distance $\Delta s_{i}$ to node $x_{i}$ can be calculated as

(3) $$\Delta s_{i}=v_{\max}\cdot t_{i}.$$

It is obvious that if task on node $x_{i}$ needs extra time to accomplish, then every visit from other nodes to node $x_{i}$ will be delayed by $t_{i}$, thus we can get the universal distance matrix $S_{d}$ by adding $\Delta s_{i}$ to the *i*th column of the distance matrix *S* where the *i*th node needs extra time. Note that we assume the time delay on the depot node is 0, so the first column of the matrix will stay the same as *S*.

(4) $$S_{d}={\left(s_{ij}^{d}\right)}_{\left(N+1\right)\times \left(N+1\right)}=\left[\begin{array}{ccccc} s_{11} & \ldots & s_{1i}+\Delta s_{i} & \ldots & s_{1\left(N+1\right)}+\Delta s_{N+1}\\ \ldots & \ldots & \ldots & \ldots & \ldots \\ s_{i1} & \ldots & s_{ii}+\Delta s_{i} & \ldots & s_{i\left(N+1\right)}+\Delta s_{N+1}\\ \ldots & \ldots & \ldots & \ldots & \ldots \\ s_{j1} & \ldots & s_{ji}+\Delta s_{i} & \ldots & s_{j\left(N+1\right)}+\Delta s_{N+1}\\ \ldots & \ldots & \ldots & \ldots & \ldots \\ s_{\left(N+1\right)1} & \ldots & s_{\left(N+1\right)i}+\Delta s_{i} & \ldots & s_{\left(N+1\right)\left(N+1\right)}+\Delta s_{N+1} \end{array}\right].$$

### 2.2. Problem Modeling with Temporal and Balanced Constraints

In this section, we formulate a 3D VRP model for finding the best routes for a group of UAVs. In both scenarios aforementioned, UAVs need to be assigned to target nodes, visit them and then return to the depot. Since we can convert the task execution time into universal distance, we can simplify the model by only considering the universal distance constraints. We define decision variable $x_{ij}^{u}=1$ if UAV *u* travels from node *i* to node *j*, otherwise $x_{ij}^{u}=0$. To balance the flight among different UAVs, as well as to satisfy the temporal resolution in some application scenarios aforementioned, the objective is to minimize the flight travelled by the UAV with the longest universal distance. The model can be presented as follows:

(5) $$minimize\left\{\underset{u=1}{\max^{M}}\left\{\sum_{i=1}^{N+1}\sum_{j=1,j\neq i}^{N+1}x_{ij}^{u}\cdot s_{ij}^{d}\right\}\right\},$$

subject to

(6) $$\sum_{j=1}^{N+1}\sum_{u=1}^{M}x_{ij}^{u}=1,\;i=2,\ldots ,N+1,$$

(7) $$\sum_{i=1}^{N+1}\sum_{u=1}^{M}x_{ij}^{u}=1,\;j=2,\ldots ,N+1,$$

(8) $$\sum_{i=1}^{N+1}x_{1i}^{u}=1,\;\forall u,$$

(9) $$\sum_{i=1}^{N+1}x_{i1}^{u}=1,\;\forall u,$$

(10) $$\sum_{i\notin S}\sum_{j\in S}x_{ij}^{u}⩾1,\;\;\forall S\subset V\backslash \left\{x_{1}\right\},\left|S\right|⩾2,\forall u,$$

(11) $$\sum_{i=1}^{N+1}\sum_{j=1,j\neq i}^{N+1}x_{ij}^{u}\cdot s_{ij}^{d}⩽s_{\max},\;\forall u,$$

(12) $$x_{ij}^{u}=\left\{\begin{array}{c} 1,\;\mathrm{if}\;\mathrm{UAV}\;u\;\mathrm{travels}\;\mathrm{from}\;\mathrm{node}\;i\;\mathrm{to}\;j;\\ 0,\;\mathrm{otherwise}. \end{array}\right.$$

$$i\neq j,i,j\in \left\{1,2,\ldots ,N+1\right\},u\in \left\{1,2,\ldots ,M\right\},$$

where *N* is the total number of target nodes, *V* is the set of nodes including the depot, and *M* is the number of UAVs, $s_{ij}^{d}$ is the universal distance from node *i* to *j*. Objective function (5) ensures that the optimization objective is to minimize the flight traveled by the UAV with the longest universal distance, thus to balance the flights and meet the temporal resolution requirements. Constraints (6) and (7) ensure that every target node will only be visited once in a round. Constraints (8) and (9) mean that for every UAV, it can only start its flight from the depot and end it in the depot. Constraints (10) are used to prevent subtours, which are degenerate tours formed between intermediate target nodes and not connected to the depot node, these constraints are also named as subtour elimination constraints (SECs) [26]. Constraints (11) ensure that for each UAV, the total flight distance would not exceed its maximum flight constraint.

### 2.3. Feasible Solution Representation and Generation

A feasible solution for a group of UAVs traversing the target nodes can be expressed by an array which elements carefully arranged. With the target scheduling and path planning model converted into an analogy of TSP problem, as shown in Figure 2, we can use a similar array to that of TSP, which consists of all target nodes and all virtual depot nodes to express a feasible solution, except that the path among all virtual depot nodes are unacceptable. To achieve this goal we can either set the distances among all virtual depot nodes to infinity or directly insert virtual depot nodes into the target nodes array.

**Figure 3:**
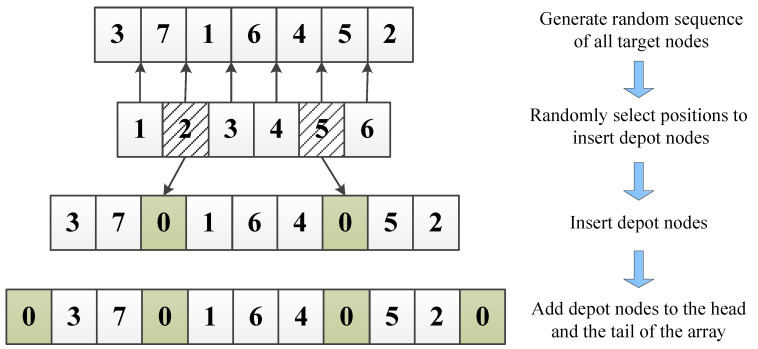
Basic procedure for generating a feasible solution. UAV number M = 3, target nodes number N = 7.

Figure 3 shows the basic procedure to generate a feasible solution. In this case, the UAV number M=3 and the target nodes number N=7. We use 0 to denote the depot node. First, we need to randomly arrange all the target nodes in an array, then randomly choose M−1 positions from N−1 of all to insert depot nodes 0, finally, we need to add starting and ending nodes 0 to the head and the tail of the array. Note that although the final feasible solution array seems like a sequence, different UAVs shall start their flights simultaneously. In this case, for UAV 1, the visiting order is 0→3→7→0, for UAV 2, the order is 0→1→6→4→0, for UAV 3, the order is 0→5→2→0. The three UAVs shall start from the depot at the same time and then return to the depot after every assigned node being visited exactly once.

We can see some characteristics of a feasible solution from Figure 3: the length of the array is N+M+1, the head and tail of the array are always 0s, and it is impossible for two virtual depot nodes to appear in the array consecutively. Algorithm 1 provides a generic approach to decide whether a randomly permutated solution makes sense, the function returns true if the input array is a feasible solution, otherwise returns false. For the efficiency of the algorithm, we do not verify constraints (11) here. On the one hand, usually, we can estimate the maximum distances for single UAV through average allocation strategy before the assignment is executed. On the other hand, due to the intrinsic attributes of the model, maximum value of terms on the left side of inequality constraints (11) will continue to decrease with the iteration of algorithms, which will be detailed in subsequent sections, so basically constraints (11), can be verified at the end of each algorithm.
**Algorithm 1: Function***Feasibility*()
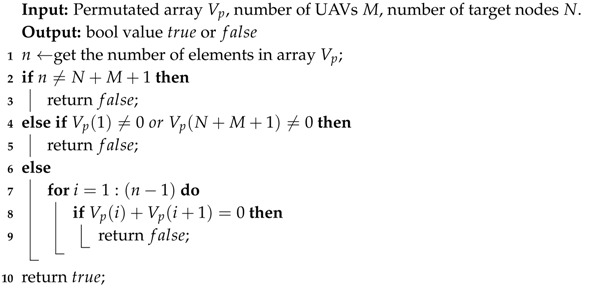


## 3. Algorithms Analysis and Design

### 3.1. Analysis and Adaptation of DE Approach

The DE algorithm introduced by Storn and Price [27] was originally designed to deal with continuous variables. When dealing with mixed discrete optimization problems with both continuous and discrete variables, DE usually represents all intrinsic parameters as floating-point values and quantifies discrete variables to the nearest permissible integer. It has been proved that DE can solve generalized combinatorial optimization problems [28].

Canonical DE begins with a randomly initiated population of NP D-dimensional vectors, also called individuals, which encode the candidate solutions to the problem, while each parameter is subject to its prescribed minimum and maximum bounds. To adapt a typical DE algorithm to the proposed UAV scheduling model, which is a combinatorial optimization problem, we use an array consists of target nodes and virtual depot nodes in a particular order to represent a feasible solution. Based on the idea of adding the difference between two vectors to another vector, we add the difference between two arrays to another array to perform the mutation. Four most frequently referred mutation strategies are adopted during this stage. The crossover operation of the classical DE method can be performed directly here due to the similar structure of the individuals. Note that in both mutation and crossover stage, the generated donor array in the mutation stage and the trial arrays in the crossover stage should all be feasible solutions, and this may need extra operations to the directly generated results. The specific method will be detailed later. The selection stage is basically the same as in original DE, which is based on the greedy principle to select the population of a new generation.

To better adapt the classical DE to the combinatorial discrete model here, we modify the original algorithm with a Delete-and-Insert strategy, namely DIDE, for the special model here. In the initialization stage of the DIDE, we adopt the procedure in Section 2.3 to quickly initialize a population of NP individuals of generation 0. The goal of the mutation operation is to generate a donor array $V_{i,G}$ from the present population. In this stage, we choose four of the most frequently adopted strategies as follows:

(13) $$DE/rand/1:\;V_{i,G}=X_{r_{1,G}^{i}}+F\cdot \left(X_{r_{2}^{i},G}-X_{r_{3,G}^{i}}\right),$$

(14) $$DE/best/1:\;V_{i,G}=X_{best,G}+F\cdot \left(X_{r_{1}^{i},G}-X_{r_{2,}^{i}G}\right),$$

(15) $$DE/rand/2:\;V_{i,G}=X_{r_{1}^{i},G}+F\cdot (X_{r_{2}^{i},G}-X_{r_{3,}^{i}G})+F\cdot (X_{r_{4}^{i},G}-X_{r_{5}^{i},G}),$$

(16) $$\begin{array}{c} DE/best/2:\;V_{i,G}=X_{best,G}+F\cdot \left(X_{r_{1}^{i},G}-X_{r_{2,}^{i}G}\right)+F\cdot \left(X_{r_{3}^{i},G}-X_{r_{4}^{i},G}\right) \end{array},$$

where $X_{r_{j}^{i},G}$ are arrays randomly chosen from the current population, $X_{best,G}$ is the optimal individual of the current population. Scaling factor *F* is a positive control parameter for scaling the difference of vectors [29].

To ensure that the generated donor array is also a feasible solution, we need to perform extra operations on the basic mutation strategies. One of the methods is to convert the parameters to floating-point numbers first for mutation, and then transform back to integer range [30]. Specifically, we first delete the virtual depot nodes from the randomly chosen $X_{r_{j},G},\;j\in \left\{1,2,\ldots ,5\right\}$, then perform one of the four mutation strategies to generate an array of floating-point numbers. To transform the elements of the array back to the integers, we convert the smallest floating-point number of the array to smallest positive integer 1, in ascending order, convert the next floating-point number to 2, the rest can be done in the same manner to get a new mutated integer array. To change the new array into a feasible solution, we need to insert the virtual depot nodes back to the array, these steps can be done as shown in Figure 3, steps 2 to 4.

In the crossover stage, the donor array mixes its components with the target array $X_{i,G}$ to form the trial array $U_{i,G}=\left[u_{1,i,G},u_{2,i,G},\ldots ,u_{D,i,G}\right]$. The DE family of algorithms uses mainly two kinds of crossover methods—exponential and binomial [27]. We adopt the binomial crossover in this stage. The scheme may be outlined as follows:

(17) $$u_{j,i,G}=\left\{\begin{array}{c} v_{j,i,G},\;\mathrm{if}\;\left(rand_{i,j}\left[0,\;1\right]⩽Cr\;or\;j=j_{rand}\right),\\ x_{j,i,G},\;\mathrm{otherwise}, \end{array}\right.$$

where the $rand_{i,j}\left[0,1\right]$ is a uniformly distributed random number, which is called anew for each *j*th element of the *i*th individual array. $j_{rand}\in \left[1,2,\ldots ,D\right]$ is a randomly chosen index, which ensures the trial array $U_{i,G}$ gets at least one element from $V_{i,G}$. As in the mutation stage, the generated array after crossover is still not a feasible solution for UAVs, the crossover operation may cause more than M+1 virtual nodes, or one target node be repeated. We employ similar operations as in the mutation stage to change the newly generated trial array into a feasible solution.

In the selection stage of DIDE, we adopt the greedy principle to determine whether the trial or the target array survives to the new generation. The selection operation can be described as

(18) $$X_{i,G+1}=\left\{\begin{array}{c} U_{i,G},\;if\;f\left(U_{i,G}\right)⩽f\left(X_{i,G}\right),\\ X_{i,G},\;if\;f\left(X_{i,G}\right)<f\left(U_{i,G}\right), \end{array}\right.$$

where f(X) is the objective function to be optimized, as Formula (5) describes. Therefore, if the new trial array yields a better or equal result of the objective function, i.e., has smaller or equal maximum universal distance, it replaces the corresponding target array in the new generation, otherwise, the target array enters the new population.

### 3.2. Analysis and Adaptation of GA Approach

GA, as a widely used meta-heuristic algorithm for solving the large-scale optimization problem, has been proven efficient for solving TSP problems [31,32]. The basic process of GA is generally composed of three steps. The first step is to select individuals from the current population based on prescribed rules, the second step is to do crossover operation on selected individuals to generate new offspring, and the third step is to randomly perform mutation technique on individuals to finally form a new generation.

The selection strategy is a critical step for the efficiency of the GA algorithm and avoiding premature convergence. Popular strategies of selection include roulette wheel, tournament, and elitist preservation strategy. Previous studies by Chudasama et al. and Razali et al. revealed in their work through comparative performance that elitism and roulette wheel methods are all efficient in their particular TSP instances, respectively. Considering that the model here is not exactly the same as TSP and the structure of a feasible solution is much different from that of TSP, we take these previous works as a reference, and combine the roulette wheel and elitist preservation method together in the proposed Modified Genetic Algorithm (MGA).

In the proportional roulette wheel strategy, individuals are selected with a probability that is directly proportional to their fitness values. When spinning the roulette wheel to select a parent, the wheel is partitioned into segments which amount corresponds to the number of individuals to be selected, and size of segments proportional to their fitness values, thus fulfilling the purpose of survival the fittest. Use $f_{i}$ to denote the fitness value, which is the reciprocal of the objective function evaluated in the individual *i*, the selection probability $p_{i}$ for individual *i* is defined as,

(19) $$p_{i}=\frac{f_{i}}{\sum_{j=1}^{n}f_{j}}.$$

For the convenience of computer simulation, we need to compute the accumulative selection probability $p_{i}^{ac}$ of each individual the population as,

(20) $$p_{i}^{ac}=\sum_{j=1}^{i}p_{j}.$$

To simulate the process of roulette wheel selection, we randomly generate a floating-point number $rand_{i}$ on [0, 1], and find the smallest value of $p_{i}^{ac}$ that is larger than $rand_{i}$, then the corresponding individual that has accumulative selection probability $p_{i}^{ac}$ is selected as a parent. The process of roulette wheel selection is described in Algorithm 2.
**Algorithm 2:** Roulette Wheel Selection
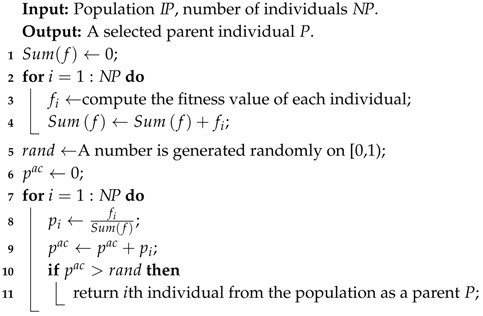


The basic idea of elitist preservation strategy is to copy the individual which is fittest directly to the next generation, rather than select all individuals based on certain probability and perform crossover and mutation operations afterward. The advantage of this strategy is that the currently fittest individual of the population would not be lost or sabotaged during selection, crossover, and mutation. The elitist preservation strategy has prominently contributed to the global convergence performance of canonical GA [33,34]. In the proposed MGA, which is adapted for the problem here, we adopt the elitist preservation strategy in both selection and mutation stage, i.e., we preserve the fittest individual of the current population in these two stages, protecting it from being sabotaged by crossover or mutation operations.

**Figure 4:**
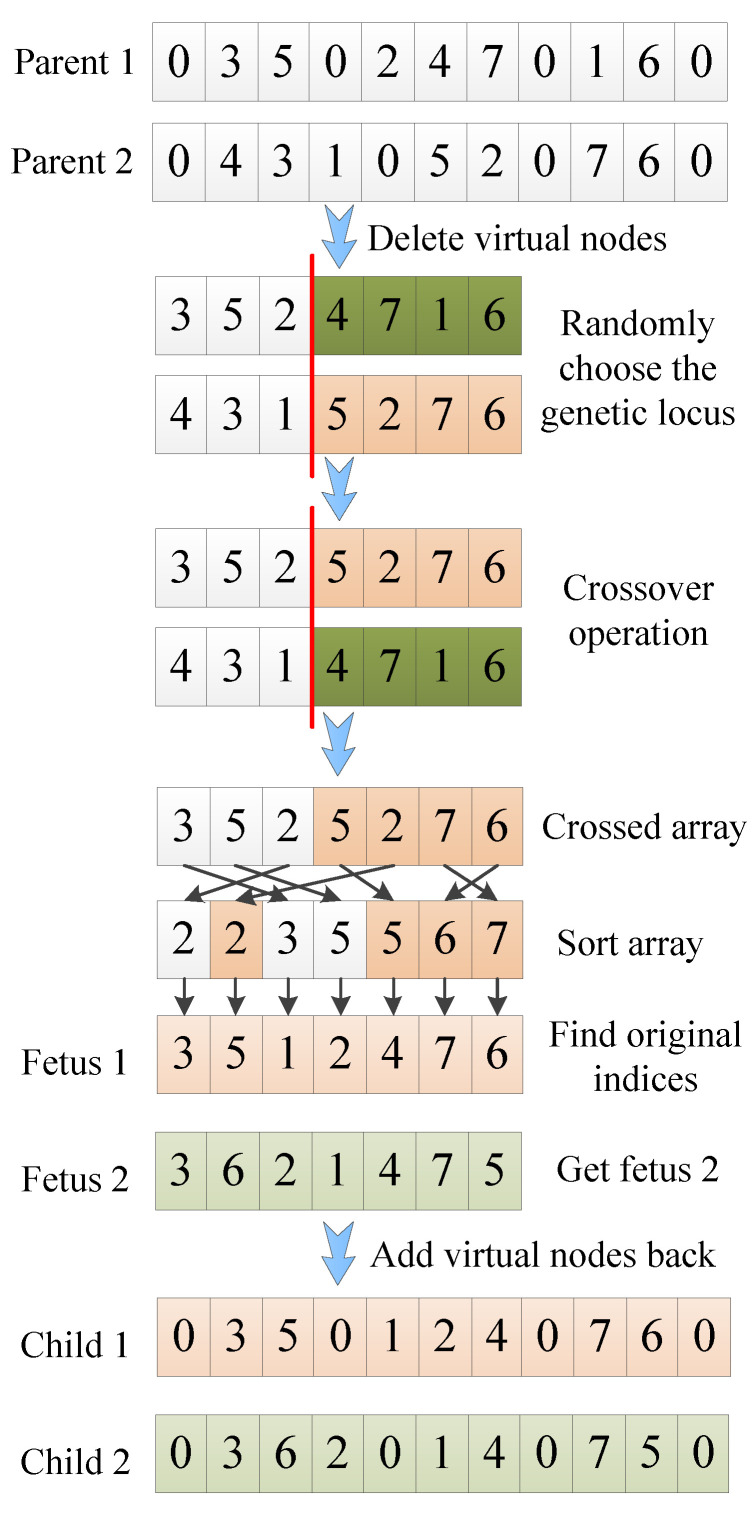
Process of crossover operation of the Modified Genetic Algorithm (MGA).

The crossover operation of MGA consists of the following steps. First, delete all virtual nodes of the two selected parent individuals. Second, randomly choose the genetic locus on parents to perform crossover, sort elements of crossed array in ascending order, and find the sorted elements’ indices in the original array to denote target nodes, thus generate two fetuses. Finally, insert virtual nodes back to previously generated fetuses to produce two offspring. The crossover process is illustrated in Figure 4.

The mutation operation is necessary for exploring the solution space. To do this we need to set the mutation rate $rate_{m}$ first, then for every individual except the elitist one, a random number $rand_{i}$ between interval [0, 1] is generated and compared with the mutation rate, if $rand_{i}$ is less than or equal to $rate_{m}$, then perform mutation on this individual, here we randomly choose two target nodes and swap them, thus generate a new solution through mutation. In every iteration, since we add two children solutions to the population after crossover, we need to delete the two worst solutions from the population based on the greedy principle at the end of each iteration.

### 3.3. Improved Simulated Annealing Approach

SA algorithm is another meta-heuristic method that has been proven effective in combinatorial optimization [35,36]. As the UAV path planning related problems and TSP problem can be categorized into combinatorial optimization, SA is also widely employed in solving these problems [20,21,23].

The requirements for implementing the classic SA algorithm are the solution representation, a method to calculate the internal energy of the solutions, an initial temperature $T_{0}$, final temperature $T^{\prime }$, and cooling factor μ. By matching the function value to the energy, SA imitates the minimization process of nature. The energy decreases are always acceptable, whereas the energy increments are accepted under certain conditions: an evenly distributed number between interval [0, 1] is less than an exponential term, and the probability of accepting less suitable solutions decreases with the reduction of temperature.

The difference between SA and the other two meta-heuristic algorithms is that SA is not a population based method, essential operation for SA is defining a proper rule to generate neighboring solution $X^{\prime }$ from current solution *X*. To efficiently generate neighboring feasible solutions, we propose a Swap-and-Judge strategy.

**Figure 5:**
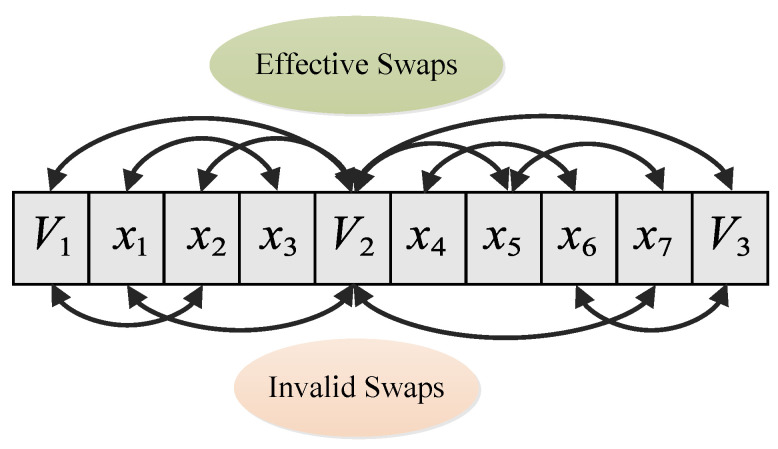
An example of effective swaps and invalid swaps.

The basic idea is of Swap-and-Judge strategy is to generate feasible solutions more efficiently without damaging the ability to explore the solution space. To do this, for a current solution, we directly choose two elements, including target nodes and virtual nodes, to do the swap operation. Figure 5 shows a possible example of effective swaps and invalid swaps in a scenario with 2 UAVs, which result in 3 virtual nodes $V_{1}$, $V_{2}$, and $V_{3}$, and 7 target nodes, $x_{1}$ to $x_{7}$. As shown in the figure, this operation may also lead to invalid swaps such as $\left\{V_{1}↔x_{2}\right\}$ and $\left\{x_{1}↔V_{2}\right\}$, although with a lower probability compared with mutation and crossover operations in previously mentioned algorithms. To ensure the feasibility, the judge function in Algorithm 3 is called here, repeat the random choosing operation if the judge function returns false until the result is true, and then do the swap operation on the current solution to generate the neighboring solution. The process of swap-and-judge strategy is detailed in Algorithm 4.
**Algorithm 3: **Function ValidityJudge()
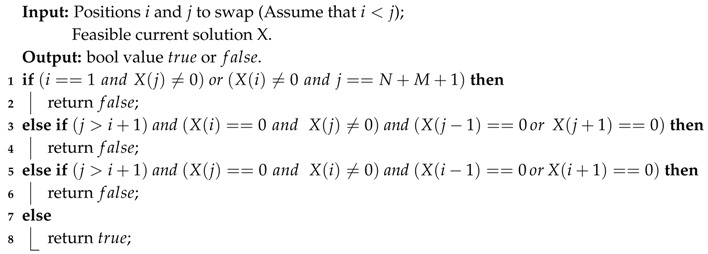

**Algorithm 4:** Swap-and-Judge Strategy for SA
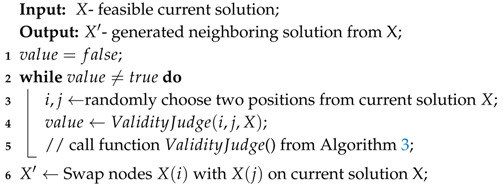


Algorithm 5 provides the basic procedure of the Swap-and-Judge Simulated Annealing (SJSA) algorithm. To determine whether the neighboring solution should replace the current one, we adopt the Metropolis algorithm [37]. As a comparison, the Delete-and-Insert method in Section 3.1 is also considered an alternative strategy to improve SA. If we replace the neighboring solution generation strategy with the Delete-and-Insert method, i.e., delete all virtual nodes from the current solution before performing the swap operation, and then add virtual nodes back, we get Delete-and-Insert Simulated Annealing (DISA) algorithm.
**Algorithm 5:** SJSA
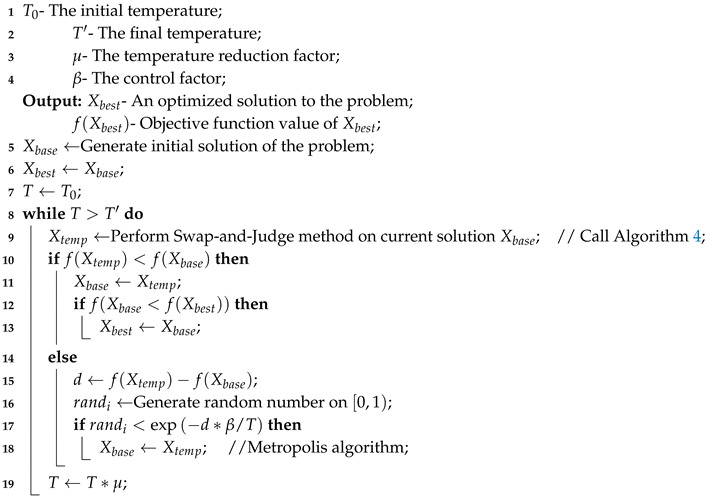


## 4. Experiments and Results

In this section, we evaluate the performance of DIDE, MGA, DISA, and SJSA in a number of randomly generated scenarios. For every proposed meta-heuristic algorithm, we validate its efficiency by several indexes including mean objective function value, standard deviation, and run time. For each type of algorithm, we also evaluate its performance under different alternative strategies or parameter settings. Especially, for SJSA, we compare its performance with the exact algorithm CPLEX to verify the efficiency and accuracy of SJSA. CPLEX 12.6 is chosen as the exact solver. All other experiments are conducted by MATLAB software on Windows 10 operating system, with Core i7-6500U 2.60GHz CPU, 8GB memory.

**Figure 6:**
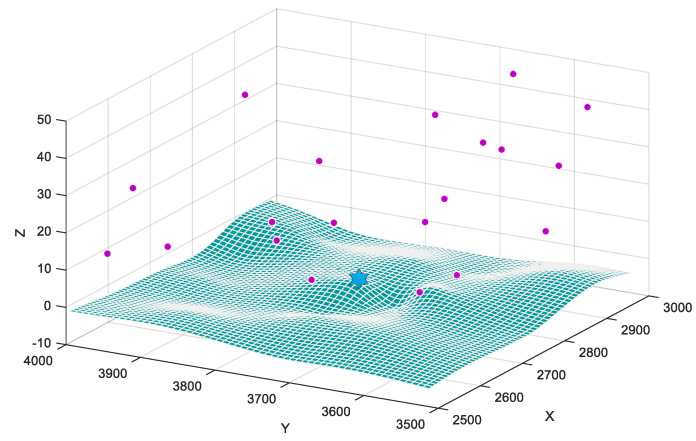
Simulation scenario.

Figure 6 shows a typical scenario in which the simulation is supposed to be applied. The blue star and purple dots represent the depot for UAVs and target nodes, respectively. All of them are distributed to a 3D space with the coordinates constrained within [2500, 3000] unit distance for the X-axis, [3500, 4000] for the Y-axis, and [0, 50] for the Z-axis. The Z-axis is stretched to better show the 3D distributions of target nodes. We assume that the terrain is basically flat for flight, and the small number of obstacles can be avoided through techniques such as light detection and image processing automatically [38]. In a mission of joint reconnaissance, multiple UAVs are supposed to visit all of the target nodes and return to the depot with a temporal resolution, i.e., they should return as a whole with the least delay possible. The three-dimensional coordinates of depot and target nodes are already known, and the extra time some nodes may need to accomplish the task is estimated in advance, therefore we can get the universal distance matrix as in Section 2.1. To better validate the effectiveness and efficiency of all proposed meta-heuristic algorithms, we create 6 scenarios A-F with a different number of target nodes and UAVs, run each type of algorithm under the same scenario to compare their performances.

### 4.1. Performance Analysis of DIDE

The results of DIDE performance with different mutation strategies are shown in Table 1. The generation and number of population of DIDE are set to Generation = 500, NP = 50. Especially, the scale factor *F* and the crossover rate Cr are configured to 0.75 and 0.45, respectively, through multiple practical simulations.

For each mutation strategy, we run the algorithm 100 times in each scenario before we collect and analyze the statistics. Run time index is obtained by dividing the total time of 100 runs by 100. For scenario A, although not prominent, strategy “best/1” appears to have a dominant advantage over other strategies on each evaluation index. For scenario B, C, and D, there is not much difference between the four strategies, although “rand/2” strategy performs better on run time index in scenario B and C, the other three strategies “rand/1”, “best/1”, and “best/2” have the best mean objective function value in scenario D, C, and B, respectively. For scenario E and F, strategy “rand/2” always acquires best mean objective function values of solutions, whereas strategy “rand/1” always gets the least run time in scenarios D, E, and F.

Although some strategies perform better in specific scenarios, the overall performances of the four strategies are not significantly different in statistics. The possible explanation for this phenomenon is that the mutation operation of DE is essentially a shuffling generator, as well as the subsequent conversion operation to make the mutated arrays to feasible solutions. Whatever the mutation strategy is, the generated donor array after the conversion operation would not be intrinsically different under these strategies.

### 4.2. Performance Analysis of MGA

As GA and DE are all population-based meta-heuristic methods, for the sake of fairness in comparison, we set the generation and number of population of MGA to Generation = 500, NP = 50, the same as in DIDE. The simulation scenario settings are also the same as in former experiments. Extensive simulations show that the mutation rate $Rate_{m}$ has a significant impact on the outcome of MGA for the UAV scheduling problem. To better show the impact of the mutation rate, we evaluate the performance of MGA in each scenario under different mutation rates 0.15, 0.35, and 0.55. The results are detailed in Table 2.

For each mutation rate $Rate_{m}$, we run the algorithm 100 times in each scenario before we collect and analyze the results. We also adopt roulette wheel selection and elitist preservation strategies in every case. Statistics in Table 2 indicate that MGA with a higher mutation rate performs better in finding solutions with the smaller objective function value. Through scenario A to F, MGA with mutation rate 0.55 always has the minimum mean objective function values and standard deviation values. These two indexes are prominently better than the other two options, although the run time index is slightly longer than the other two options. MGA with mutation rate 0.15 has the shortest run time in each scenario, the difference of run time among three options are not significant but pretty steady through different scenarios.

The phenomenon in Table 2 provides interesting inspirations. We know from the detailed process of the MGA method that the mutation strategy is essentially a way to explore the solution space more thoroughly, otherwise the MGA would only generate two other solutions for each generation through crossover operation. To make better use of the population, a higher mutation rate is needed to explore neighboring solutions from the current population. On the other hand, a higher mutation rate also needs more computation resources to generate new solutions. In practical application, as the run time increase is less than a linear increase in each scenario, we can configure the mutation rate to achieve a balance between the ideal mean function value and run time.

### 4.3. Performance Analysis of SJSA and DISA

We run DISA and SJSA under the same scenarios A to F as before. For both algorithms, the parameters are set to $T_{0}$ = 1000 as initial temperature, μ = 0.99 as the reduction factor, and β = 100 as the control variable. Different from former algorithms, Simulated Annealing is not a population-based algorithm, and normally only one new solution is generated in each iteration of the algorithm. To ensure the fairness of the comparison, we configure the final temperature $T^{\prime }$ for DISA and SJSA to generate basically as many individuals as former population-based methods. We run each algorithm for 100 times in each scenario as before to evaluate the results. The performance of DISA and SJSA are detailed in Table 3.

The statistics clearly show that the performance of SJSA is dominantly better in each case by all indexes than DISA, especially in scenarios C to F. One of the obvious explanation is that the swap-and-judge operation of SJSA is more efficient than the delete-and-insert operation of DISA, especially when the number of UAVs is small, in which case the ratio of virtual nodes to the target nodes is small, therefore the times of validity judge operations can be largely reduced. As SJSA can directly generate a feasible solution from the current one without time-consuming delete and insert operation, it shows higher efficiency than DISA. As the scale of the problem increases, the gap between the optimized results by the two methods tends to widen. Meanwhile, the time consumption of SJSA stays at a pretty low level and has a slow growth rate compared with DISA, which is a good sign that SJSA can be more competitive in related large scale optimization problems and their generalized forms.

### 4.4. Comparative Study

#### 4.4.1. Comparison with CPLEX

To verify the optimization performance of the proposed SJSA, which is the best among all of the algorithms proposed before, we compare it with the exact algorithm, CPLEX, to find the gap between the satisfactory solution generated by SJSA and the optimal solution obtained by CPLEX. From the model and constraints in Section 2.2, we can see that the computational complexity of the problem is very high. Taking constraints (10) for example, for any nodes set *V* containing N+1 nodes including the depot node, there are as many as $2^{N}$ different subsets S for $V\backslash \left\{x_{1}\right\}$, which means a pretty high time complexity only for constraints (10), not to mention other constraints. This will cause the amount of calculation to increase exponentially when the number of target points increases. For the convenience of comparison, we configure a set of test scenarios S1 to S8 with the relatively small number of target nodes to conduct the comparative experiments between SJSA and CPLEX. For each test scenario, we run the SJSA for 20 times and calculate the mean values of the objective function and run time for comparison with the optimal value obtained by CPLEX, and the results are detailed in Table 4.

From the statistics in Table 4, we can see that the time consumption of CPLEX increases explosively with the increase of target nodes number. When the number of target nodes increases from 8 to 15, which is less than doubled, the time consumption increases from 33.39 s to nearly 8 h. For scenario S6, which with only 5 UAVs and 13 target nodes, the running time has exceeded an hour, whereas SJSA can find the near-optimal solution in no more than 1 s and the average gap with the optimal solution is less than 2%. The time consumption of SJSA grows quite slowly in finding near-optimal solutions with the increase of target nodes number and UAV number, a phenomenon that can also be observed in previous experiments. At the same time, the average gaps with the optimal solution obtained by CPLEX in all these test scenarios are kept within 3%. It is worth mentioning that in 20 runs for all of the eight scenarios, there are always several times in each scenario when SJSA can get the optimal solution. Although due to computer hardware limitations, we can not conduct experiments on CPLEX with much more target nodes for comparison with SJSA, we believe that the statistics in the table can testify to some extent that SJSA is efficient in finding near-optimal solutions for the problem here, especially for large-scale optimization problem with a large number of target nodes.

#### 4.4.2. Comparison with Proposed Meta-Heuristic Algorithms

We further conduct the comparative study by analyzing the performances of different proposed algorithms in each scenario. For algorithm DIDE, we always choose the best mean value and its corresponding standard deviation from the four strategies. As for algorithm MGA, we choose the best mean value and its corresponding standard deviation from different mutation rate values, i.e., choose the case in which the mutation rate is $Rate_{m}=0.55$. Together with DIDE and MGA, we make a parallel comparison between DISA and SJSA, as these two algorithms have very different strategies and prominent differences.

**Figure 7:**
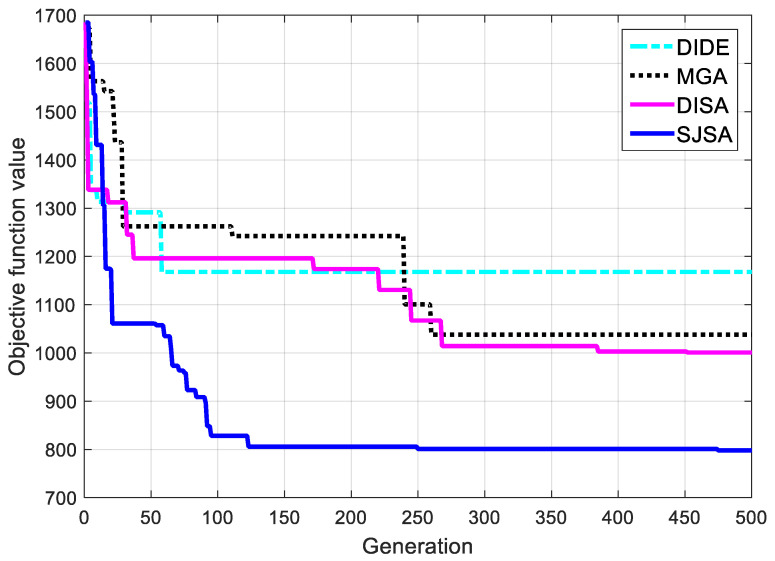
Convergence comparison in scenario C.

The convergence curves of the four algorithms in scenario C are illustrated in Figure 7. It is obvious that the convergence effect of the proposed SJSA is better than other algorithms. The SJSA continually converges to better objective function without being trapped by local optimum and attains its optimal value in iteration 475. The DISA approach also continually converges but with a slower speed and stays at its optimal value in iteration 452. The MGA performs better than DIDE in terms of continuous convergence, but the convergence speed is slower compared with SA-based algorithms. The DIDE approach, on the other hand, has a very slow speed of convergence and stays at a relatively high objective function value at the end of the iteration.

**Figure 8:**
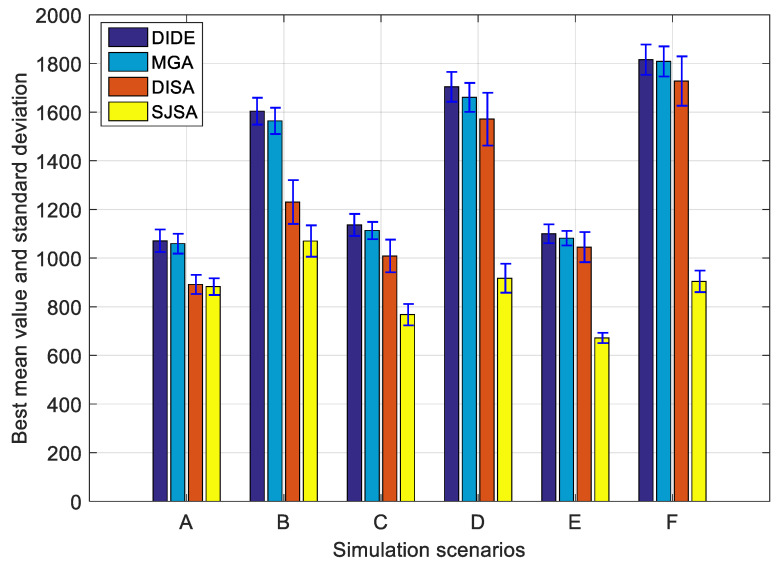
Performance comparison among scenarios.

Figure 8 shows the best mean objective function values and corresponding standard deviations of different algorithms in scenarios A to F. For DIDE, we always choose the best results from the four strategies, and similarly, we choose the results of MGA from $Rate_{m}=0.55$, which always gets better results than other choices. In terms of mean function value, the proposed algorithm SJSA outperforms DIDE, MGA, and DISA by around 38.17%, 37.10%, and 30.26%, respectively. DISA, on the other hand, outperforms DIDE and MGA by around 11.34% and 9.81%, respectively. Whereas MGA is always slightly better than DIDE in each scenario. Two reasons may explain why DISA and SJSA outperform DIDE and MGA. (1) DIDE and MGA are all population-based algorithms, whereas the two SA-based algorithms are not. The transformation operations such as mutation and crossover are essentially a shuffle mechanism, which is of no directivity. Therefore, changing the mutation strategy, as in DIDE, would have subtle influence on the outcome of the shuffling results. This may also explain why the four strategies perform very close. As for MGA, increasing the mutation rate can surely increase the probability of generating new solutions, thus exploring the solution space more thoroughly, but this can also increase the algorithm running time. (2) DISA and SJSA, as SA-based algorithms, have the advantage of accepting inferior solutions with a changing probability, this can help them to a great extent avoid falling into local optimality at early stage.

**Figure 9:**
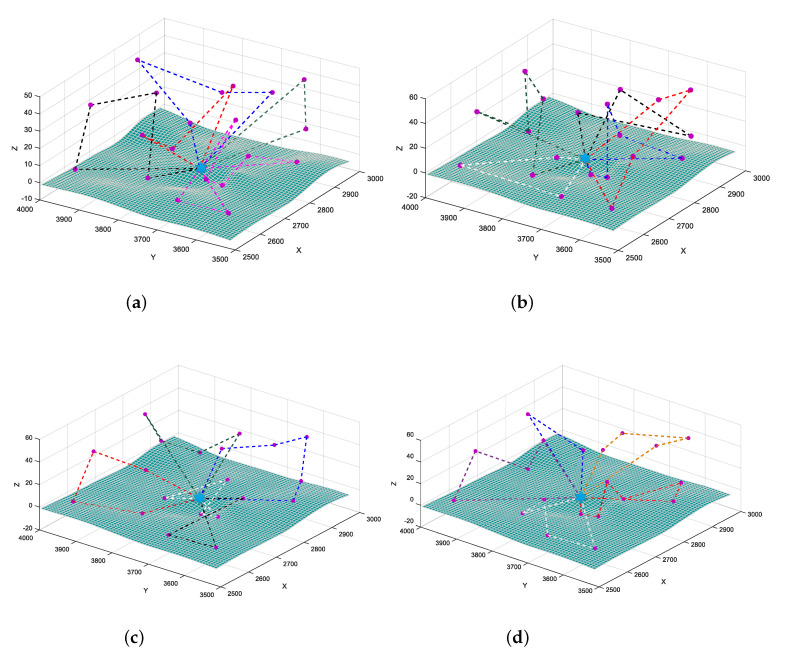
Comparison of optimized results by different methods in scenario C (M = 5, N = 20). (**a**) Optimized result of DIDE (best/1); (**b**) optimized result of MGA ($Rate_{m}=0.55$); (**c**) optimized result of DISA; (**d**) optimized result of SJSA.

We further graphically display the optimized results by different methods in Figure 9. Take scenario C (M = 5, N = 20) as an example, we run four algorithms and display the scheduling results. The blue star represents the depot node, whereas the purple dots represent the target nodes that need to be visited within the temporal resolution. Dotted lines with different colors represent task assignments and execution orders for different UAVs. For DIDE, we adopt the best/1 strategy as an example, and for MGA, we configure the mutation rate to $Rate_{m}=0.55$. Four algorithms all generate feasible solutions for 5 UAVs, the scheduling result generated by SJSA is better statistically, and also visually contains less crossover among flights than other methods, which make it easier for UAVs to perform flight missions. Scheduling results by DISA and SJSA also look clearer than by the former two methods, which means a single UAV is basically responsible only for a small discrete area of its own task, and there are fewer overlaps among different task areas.

## 5. Conclusions and Future Work

In this paper, to solve the problem of task assignment and path planning for multiple UAVs in practical missions, an algorithm, namely SJSA is proposed based on comparison with other meta-heuristic algorithms. By adding the virtual nodes into the 3D VRP model, we find a convenient way to generate feasible solutions suitable for heuristic algorithms. For the balanced distribution of tasks among UAVs, the concept of universal distance matrix is proposed to simplify the computational work. The task assignment and path planning model is therefore established with multiple constraints considered. To efficiently find the suboptimal solution for the model, we also study and modify population-based evolutionary algorithms such as DE and GA, and designed the efficient SJSA based on SA. Experimental and comparative studies reveal that the proposed SJSA is efficient in finding near-optimal solutions compared with the exact algorithm CPLEX, and also competitive in comparison with proposed meta-heuristic algorithms, the results also suggest that the population-based algorithms may not suitable for this discrete combinatorial optimization problem.

In our future work, we will focus on the path planning for UAVs in a complex environment, which is challenging for the computational resource and efficiency of algorithms. Besides, we will also extend our study to the multiobjective optimization in some special application scenarios, while taking more inherent flight characteristics of UAV into account.

## Figures and Tables

**Table 1:** Performance comparison of Delete-and-Insert Differential Evolution (DIDE) with different mutation strategies.

Scenarios	Rand/1			Best/1			Rand/2			Best/2		
	Mean	Std	Runtime (s)	Mean	Std	Runtime (s)	Mean	Std	Runtime (s)	Mean	Std	Runtime (s)
A (M = 3, N = 15)	1076.34	46.85	1.95	1070.93	46.29	1.92	1072.53	48.83	2.03	1074.29	46.29	1.92
B (M = 3, N = 20)	1606.26	52.18	1.98	1605.10	49.51	2.07	1607.39	50.70	1.92	1603.97	55.16	2.10
C (M = 5, N = 20)	1142.17	45.37	2.31	1136.44	45.06	2.21	1142.63	38.28	2.20	1143.04	43.14	2.40
D (M = 5, N = 30)	1703.88	61.02	2.33	1709.31	57.92	2.35	1713.38	56.31	2.60	1704.33	67.27	2.70
E (M = 10, N = 30)	1103.24	37.76	3.16	1103.81	41.20	3.25	1099.82	38.53	3.82	1100.67	34.33	3.67
F (M = 10, N = 50)	1823.65	56.92	3.50	1830.15	58.47	3.79	1815.65	62.33	3.91	1816.12	61.96	3.53

**Table 2:** Performance comparison of MGA with different mutation rates.

Scenarios	${\mathrm{Rate}}_{m}=0.15$			${\mathrm{Rate}}_{m}=0.35$			${\mathrm{Rate}}_{m}=0.55$		
	Mean	Std	Runtime (s)	Mean	Std	Runtime (s)	Mean	Std	Runtime (s)
A (M = 3, N = 15)	1085.27	43.71	1.11	1063.00	44.36	1.28	1059.39	40.76	1.66
B (M = 3, N = 20)	1616.16	61.80	1.01	1582.19	56.58	1.31	1564.19	54.02	1.62
C (M = 5, N = 20)	1157.50	46.00	1.07	1127.89	40.18	1.42	1113.30	35.57	1.77
D (M = 5, N = 30)	1741.81	68.04	1.12	1696.63	60.37	1.55	1660.55	59.78	1.85
E (M = 10, N = 30)	1113.02	46.52	1.32	1094.93	40.47	1.87	1081.84	29.99	2.39
F (M = 10, N = 50)	1880.88	63.61	1.38	1842.74	63.87	1.95	1808.45	62.03	2.48

**Table 3:** Performance comparison of Delete-and-Insert Simulated Annealing (DISA) and Swap-and-Judge Simulated Annealing (SJSA).

Scenarios	DISA			SJSA		
	Mean	Std	Runtime (s)	Mean	Std	Runtime (s)
A (M = 3, N = 15)	891.71	39.32	2.17	882.64	34.50	0.19
B (M = 3, N = 20)	1230.44	89.92	2.14	1070.15	64.46	0.22
C (M = 5, N = 20)	1008.59	67.20	2.58	767.49	44.14	0.26
D (M = 5, N = 30)	1571.36	108.55	2.63	917.00	59.55	0.30
E (M = 10, N = 30)	1045.22	62.10	3.39	671.62	20.90	0.35
F (M = 10, N = 50)	1727.61	101.57	3.82	904.14	44.24	0.38

**Table 4:** Performance comparison of CPLEX and SJSA.

Test Scenarios	CPU (Sec)		Objective Function Value		Gap (%)
	CPLEX	SJSA	CPLEX	SJSA	
S1 (M = 3, N = 8)	33.39	0.21	757.37	764.14	0.89
S2 (M = 3, N = 9)	56.77	0.22	542.16	549.51	1.36
S3 (M = 4, N = 10)	215.53	0.22	671.77	686.42	2.18
S4 (M = 4, N = 11)	477.57	0.22	597.21	603.13	0.99
S5 (M = 4, N = 12)	1257.15	0.23	719.96	728.14	1.14
S6 (M = 5, N = 13)	5713.42	0.28	644.63	653.26	1.34
S7 (M = 5, N = 14)	15811.99	0.36	613.49	618.81	0.87
S8 (M = 5, N = 15)	28042.32	0.37	641.45	650.46	1.40

## Abbreviations

The following abbreviations are used in this manuscript:

DE	Differential Evolution
DIDE	Delete-and-Insert Differential Evolution
GA	Genetic Algorithm
IoT	Internet of Things
MGA	Modified Genetic Algorithm
PSO	Partical Swarm Optimization
QoE	Quality of Experience
QoS	Quality of Service
SA	Simulated Annealing
SJSA	Swap-and-Judge Simulated Annealing
SOS	Symbiotic Organisms Search
TSP	Traveling Salesman Problem
UAV	Unmanned Aerial Vehicle
VRP	Vehicle Routing Problem
